# Chondroprotective Effect of *Campylaephora hypnaeoides* Extract in Primary Chondrocytes and Rat OA Model

**DOI:** 10.3390/ijms252413391

**Published:** 2024-12-13

**Authors:** Ji Yun Jang, Seul Ah Lee, Do Kyung Kim, Sook-Young Lee, Chun Sung Kim

**Affiliations:** 1 Marine Healthcare Research and Evaluation Center, Chosun University, Wando 59146, Republic of Korea; greenbee16@naver.com (J.Y.J.); seedbank@chosun.ac.kr (S.-Y.L.); 2 Department of Oral Biochemistry, College of Dentistry, Chosun University, Gwangju 61452, Republic of Korea; seulah21@naver.com; 3 Department of Oral Biology, College of Dentistry, Chosun University, Gwangju 61452, Republic of Korea; kdk@chosun.ac.kr

**Keywords:** osteoarthritis, *Campylaephora hypnaeoides*, chondroprotective effect, rat chondrocytes, DMM

## Abstract

*Campylaephora hypnaeoides* (*C. hypnaeoides*) was extracted using fermented ethanol. The effect of fermented ethanol extract of *C. hypnaeoides* (FeCH) on chondrocyte viability was analyzed by 3-(4,5-dimethylthiazol-2-yl)-2,5-iphenyltetrazolium bromide assay, which showed no cytotoxicity at 2 mg/mL. FeCH pretreatment in IL-1β-stimulated chondrocytes significantly inhibited the accumulation of nitric oxide and prostaglandin E_2_, which was analyzed using the ELISA assay. In addition, protein expression levels of inflammatory-related factors, such as inducible nitric oxide synthase, cyclooxygenase-2, interleukin-6, tumor necrosis factor-alpha, and cartilage-degrading-related enzymes, such as matrix metalloproteinases-1, -3, and -13, and a disintegrin and metalloproteinase with thrombospondin motifs-4 and -5 were significantly decreased in IL-1β-stimulated chondrocytes pretreated with FeCH, which were analyzed using western blot analysis. In addition, as a result of analyzing the content of collagen type II (Col II) and proteoglycan through western blot analysis and alcian blue staining, FeCH pretreatment prevented the degradation of Col II and proteoglycan. It was analyzed through western blot analysis that the chondroprotective effect of FeCH may be mediated through MAPKs and NF-κB-signaling mechanisms. In an in vivo study, an osteoarthritis experimental animal model with damaged medial meniscus (DMM) was utilized and orally administered daily for 8 weeks after surgery. At the study end point, knee joints were harvested and subjected to histological analysis with safranin O staining. As a result, articular cartilage was significantly protected in the FeCH group compared to the DMM group. These results suggest FeCH as a candidate material for the development of pharmaceutical materials for the treatment or prevention of degenerative arthritis.

## 1. Introduction

Osteoarthritis (OA), the most common joint disease in contemporary society, is the inability to repair joint damage caused by stress from joint or periarticular tissue abnormalities [1]. The causes of OA are highly diverse and may include obesity, sports, genetic factors, and joint anatomical abnormalities [2]. Chondrocytes are highly specialized and, metabolically, cells in articular cartilage that perform unique functions in the production, maintenance, and repair of the extracellular matrix (ECM) but are characterized by low apoptotic activity and an inability to divide [2,3]. Therefore, maintaining healthy chondrocytes is extremely important [4,5]. Recently, excessive inflammation has been suggested as an early cause of osteoarthritis.

Chondrocytes damaged by frequent friction increase their secretion of pro-inflammation-related cytokines such as nitrite, interleukin (IL)-1β, tumor necrosis factor (TNF)-α, IL-6, and pain-related factors such as cyclooxygenase (COX-2) [2]. These factors cause excessive inflammation by increasing the inflammatory environment, ultimately disabling the function of chondrocytes, the only cells responsible for keeping cartilage healthy. The intake of functional foods increases joint health and helps maintain healthy joints. However, concerns have been raised regarding the efficacy of glucosamine and MSM, which account for 80% of the raw materials of joint health functional foods [6]. Additionally, commonly used treatments for OA—such as acetaminophen, nonsteroidal anti-inflammatory drugs, COX-2 inhibitors, and intra-articular hyaluronic acid or steroid injections—are associated with various side effects [7,8]. Therefore, this study aimed to develop safe materials that exhibit significant efficacy, providing a scientific foundation for joint health [9,10].

Sea algae contain various active components that maintain health and prevent diseases. Seaweeds are rich sources of phenolic compounds. Phenolic derivatives also exhibit a broad spectrum of biological properties, including antioxidant, anti-inflammatory, antimicrobial, anticancer, antidiabetic, and anti-obesity activities [11]. These active components have been investigated for potential use in food, cosmetic, and pharmaceutical applications [12]. *C. hypnaeoides* is an edible seaweed belonging to the red algae family, distributed along the coasts of Korea and Japan. *C. hypnaeoides* is rich in polysaccharides and has been used since ancient times [13]. Recently, it has been reported that *C. hypnaeoides* ethanol extract reduces body fat and alleviates metabolic disorders through its anti-inflammatory and antioxidant activities [12]. However, this is the only functional group reported thus far. Therefore, in this study, the chondroprotective effect and mechanism of action of FeCH were analyzed in primary chondrocytes and experimental animal models of OA.

## 2. Results

### 2.1. TSC, TPC, TFC, and TCC of CH Extracts

The TSC, TPC, TFC, and TCC values of the CH extracts are listed in Table 1. The TSC value is 166.77 ± 1.3 mg glucose/100 g dry weight, and the TPC value is 167.2 ± 1.5 mg GA/100 g dry weight. The TFC value is 59.5 ± 0.8 mg QUE/100 g dry weight, and the TCC value is 1.16 ± 0.06 mg lutein/100 g dry weight.

### 2.2. Effect of FeCH on the Cell Viability of RC

**Figure 1:**
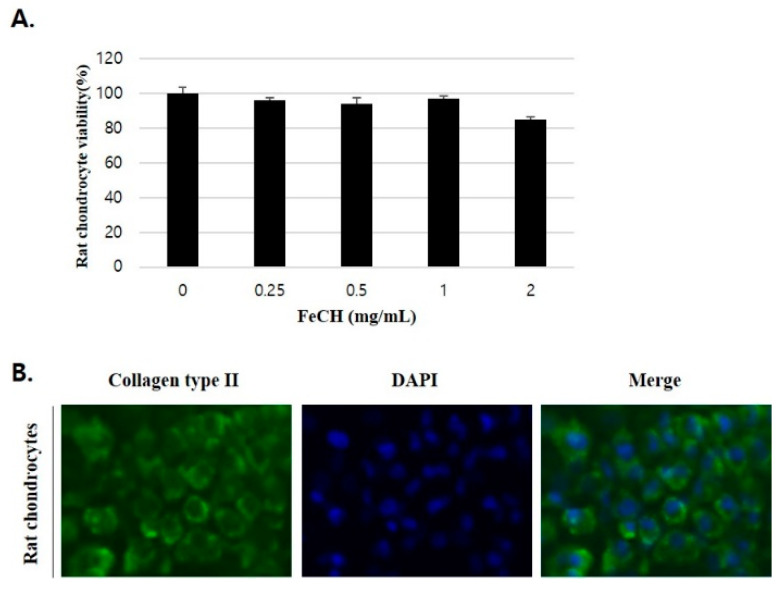
Effects of fermentation ethanol of *Campylaephora hypnaeoides* (FeCH) on primary rat chondrocyte viability. (**A**) primary rat chondrocytes were treated with FeCH (0.25, 0.5, 1, and 2 mg/mL) for 24 h, and viability was determined by MTT assay. (**B**) ICC staining of chondrocytes using collagen type II antibody after 3 days of culture (×40). Data are represented as mean ± SD of three independent experiments.

The MTT assay was performed to analyze the effects of FeCH on chondrocyte viability. Treatment with concentrations up to 2 mg/mL did not affect cell viability (Figure 1A). Immunocytochemistry was performed using an anti-collagen type II antibody to confirm that the isolated cells were chondrocytes. Collagen type II was distributed throughout the cytoplasm, suggesting that the isolated cells were chondrocytes (Figure 1B). In subsequent experiments, the extract was evaluated for efficacy at concentrations of 0.125, 0.25, and 0.5 mg/mL.

### 2.3. Inhibitory Effect of FeCH on IL-1β-Induced Expression of NO, iNOS, COX-2, COX-1, TNF-α, IL-6, and PGE_2_ in RC

**Figure 2:**
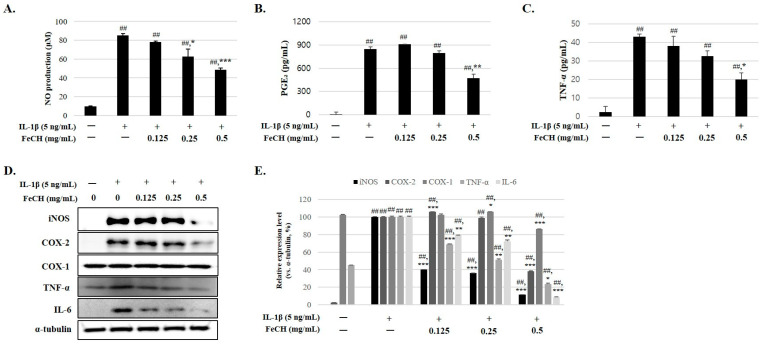
Inhibitory effects of FeCH on IL-1β-induced nitrite, PGE_2_, TNF-α, IL-6, and COX-2 in primary rat chondrocytes. Primary rat chondrocytes were pretreated with FeCH (0.125, 0.25, and 0.5 mg/mL) for 1 h, followed by IL-1β (5 ng/mL) for 24 h. Nitrite production (**A**), PGE₂ production (**B**), and TNF-α production (**C**) in the cell culture medium were determined by ELISA kit. (**D**) Expression of iNOS, COX-2, TNF-α, IL-6, and COX-1 expression was determined by western blot. (**E**) Quantitative data of (**D**) were analyzed using Image J software (version 8). Data are represented as mean ± SD of three independent experiments. α-tubulin served as an internal control. ## *p* < 0.01 vs. control; * *p* < 0.05, ** *p* < 0.01, and *** *p* < 0.001 vs. IL-1β-treated group.

The overexpression of pro-inflammatory cytokines, such as NO, iNOS, PGE_2_, and COX-2, due to inflammation is a major factor in the development of osteoarthritis. Therefore, to evaluate whether the extract has an anti-inflammatory effect, the expression changes of inflammation-related factors induced by the extract in IL-1β-stimulated chondrocytes were analyzed. Chondrocytes stimulated with IL-1β increased secretion of NO, iNOS, PGE2, TNF-α, and IL-6 compared with unstimulated chondrocytes, and protein expression was also significantly increased (Figure 2A–E), but their expression was inhibited by the FeCH in a concentration-dependent manner (Figure 2A–E). In addition, COX-1, related to gastric mucosal protective enzymes, appeared consistently regardless of whether inflammation was induced (Figure 2D). These results suggest that FeCH exerts anti-inflammatory effects.

### 2.4. Inhibitory Effect of FeCH on the Expression of ADAMTS-5, ADAMTS-4, MMP-1, MMP-3, and MMP-13 Induced by IL-1β in RC

**Figure 3:**
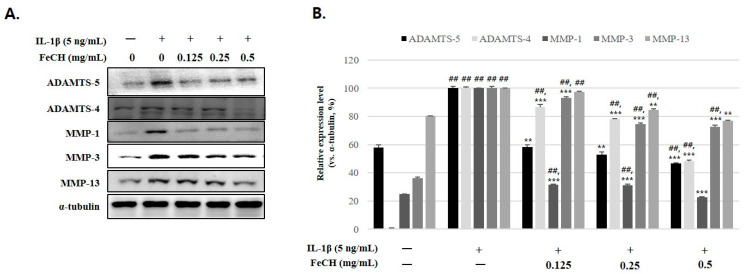
Inhibitory effects of FeCH on IL-1β-induced ADAMTS-5 and -4 and MMP-1, -3, and -13 in primary rat chondrocytes. Primary rat chondrocytes were pretreated with FeCH (0.125, 0.25, and 0.5 mg/mL) for 1 h, followed by IL-1β (5 ng/mL) for 24 h. (**A**) Protein levels of matrix-degrading enzymes (ADAMTS-5 and -4 and MMP-1, -3, and -13) was determined by western blot. (**B**) Quantitative data of (**A**) were analyzed using Image J software. Data are represented as mean ± SD of three independent experiments. α-tubulin served as an internal control. ## *p* < 0.01 vs. control; ** *p* < 0.01, and *** *p* < 0.001 vs. IL-1β-treated group.

To evaluate the effects of FeCH on the activity of enzymes that degrade the cartilage matrix, the expression of ADAMTSs and MMPs was analyzed. IL-1β significantly increased the expression of ADAMTS-5 and -4 and MMP-1, -3, and -13, but their activities gradually decreased by pretreatment with FeCH (Figure 3A,B).

### 2.5. Protective Effect of FeCH Against IL-1β-Induced Collagen Type II and Proteoglycan Degradation in RC

**Figure 4:**
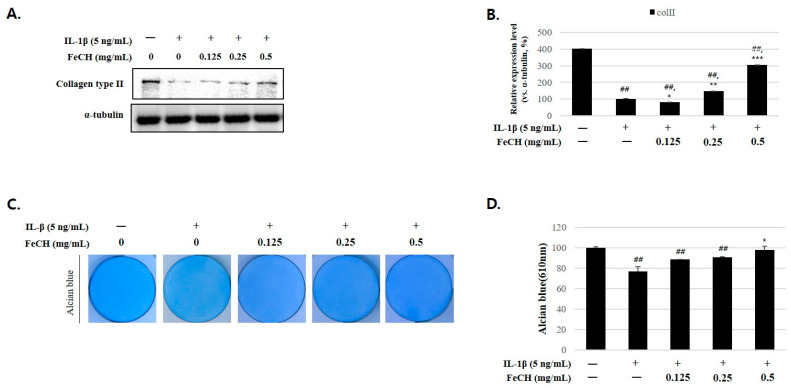
Effect of FeCH on collagen type II and proteoglycan in IL-1β-stimulated primary rat chondrocytes. Primary rat chondrocytes were pretreated with FeCH (0.125, 0.25, and 0.5 mg/mL) for 1 h, followed by IL-1β (5 ng/mL) for 24 h. (**A**) Protein levels of collagen type II degradation were determined by western blot. (**B**) Quantitative data of (**A**) were analyzed using Image J software. (**C**) Proteoglycan degradation was determined by alcian blue stain. (**D**) After dissolving alcian blue dye in 6M guanidine-HCL, the absorbance at 620 nm was measured. Data are represented as mean ± SD of three independent experiments. α-tubulin served as an internal control. *## p* < 0.01 vs. control; * p < 0.05, ** p < 0.01, and *** p < 0.001 vs. IL-1β-treated group.

In the context of cartilage maintenance, Col II and proteoglycans serve as pivotal constituents of the ECM. Although these components are typically abundant in the normal cartilage, their levels decrease with cartilage damage. Western blotting was performed to validate the inhibitory effects of FeCH pretreatment on Col II concentration, and the results showed that pretreatment with FeCH increased Col II expression in a concentration-dependent manner (Figure 4A,B). Furthermore, to confirm the protective effect of FeCH pretreatment, its influence on proteoglycan degradation was analyzed using alcian blue staining. These results indicate that IL-1β led to a reduction in proteoglycan concentrations. Pretreatment with FeCH inhibited proteoglycan degradation in a concentration-dependent manner (Figure 4C,D).

### 2.6. FeCH Exerts Chondroprotective Effects Through the Inhibition of NF-κB p65 Nuclear Translocation and MAPK Phosphorylation in RC

**Figure 5:**
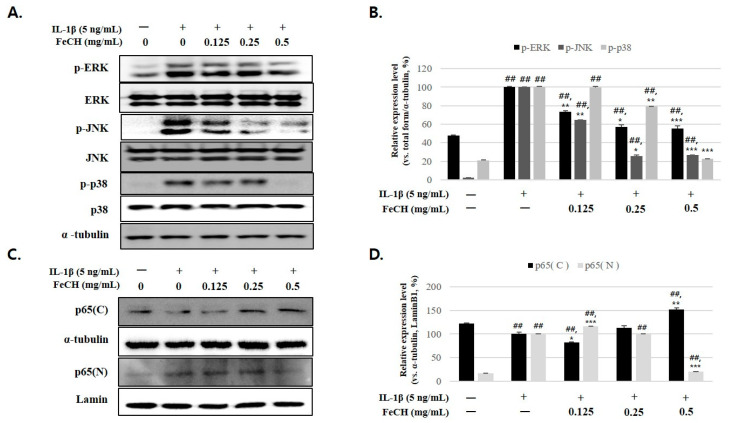
Effects of FeCH on phosphorylation of MAPKs and activation of NF-κB in IL-1β-stimulated primary rat chondrocytes. Primary rat chondrocytes were pretreated with FeCH (0.125, 0.25, and 0.5 mg/mL) for 1 h, followed by IL-1β (5 ng/mL) for 24 h. (**A**) Protein levels of phosphorylation of MAPKs were determined by western blot. (**B**) Quantitative data of (**A**) were analyzed using Image J software. (**C**) Phosphorylation levels of NF-κB p65 were determined by western blot. (**D**) Quantitative data of (**C**) were analyzed using Image J software. Data are represented as mean ± SD of three independent experiments. α-tubulin and Lamin B1 served as internal controls. *## p* < 0.01 vs. control; * p < 0.05, ** p < 0.01, and *** p < 0.001 vs. IL-1β-treated group.

To analyze the mechanism of the chondroprotective effect of 30% FeCH, the phosphorylation of MAPKs (ERK, JNK, p38) and the activity of NF-κB p65 subunit were analyzed using western blot. IL-1β phosphorylation of MAPKs, including ERK, JNK, and p38, was activated when inflammation was induced. However, pretreatment with the FeCH extract inhibited the phosphorylation of ERK, JNK, and p38 (Figure 5A,B). IL-1β induces phosphorylation of NF-κB p65, followed by nuclear translocation (Figure 5C,D). In contrast, pretreatment of chondrocytes with FeCH inhibited NF-κB p65 phosphorylation and nuclear migration (Figure 5C,D).

These results suggest that IL-1β-induced expression of cartilage degradation enzymes in RC is mediated through MAPKs and NF-κB-signaling pathways and pretreatment with FeCH in RC inhibits the signaling pathway, leading to ECM degradation.

### 2.7. The Chondroprotective Effect of FeCH in an Osteoarthritis Disease Animal Model

**Figure 6:**
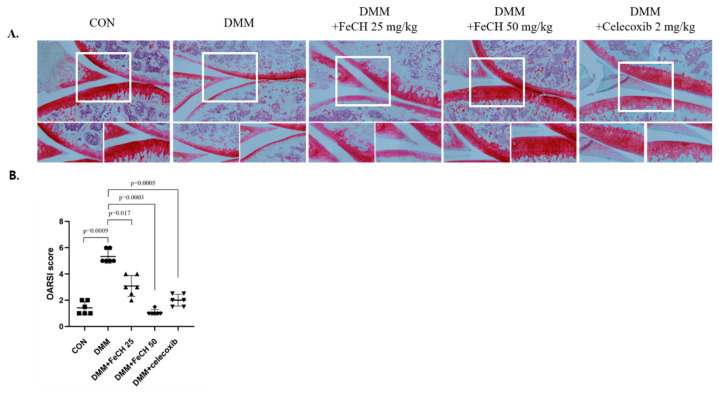
Histological evaluation of cartilage-protective effect of FeCH against cartilage degradation in a DMM model. (**A**) In vivo schemic (×100). White box indicates the enlarged area. (**B**) Rats underwent surgical destabilization of the medial meniscus (DMM). The day after the DMM surgery, rats were orally administered with FeCH (25 and 50 mg/kg) or celecoxib (2 mg/kg) daily for 8 weeks. Histological analysis of cartilage destruction was evaluated by safranin O/fast green staining.

To investigate the chondroprotective effect of the extract in a DMM-induced OA model, we evaluated the structural integrity of articular cartilage using safranin O/fast green staining and the OARSI score. Group 2 (DMM) exhibited severe osteoarthritis compared with the control group, with observed cartilage defects such as decreased proteoglycans and damage to the cartilage surface. The OARSI score for this group was 5.5 ± 0.5 (Figure 6A,B). However, the OA groups treated with 25 and 50 mg/kg extract showed less proteoglycan loss and less cartilage destruction in the articular cartilage, resulting in a decrease in cartilage defects (Figure 6A). Additionally, the blind test resulted in low OARSI scores (3 ± 1 and 1.25 ± 0.25, respectively) (Figure 6B). These results suggest that treatment with the extract inhibited OA progression in a rat OA model.

## 3. Discussion

Previously, it was thought that joint cartilage destruction and inflammation occurred due to chronic overload and damage to the joints, but osteoarthritis is now reported to be a more intricate and diverse process involving inflammation and metabolic factors [14].

CH has been consumed as food by boiling and hardening since ancient times. Murakami et al. (2022) reported that a CH ethanol extract alleviates metabolic disorders through its anti-inflammatory and antioxidant effects [12]. Although the anti-inflammatory effects of CH have been reported, their effects on inflammation-related diseases have not yet been investigated. Therefore, in this study, we investigated the cartilage-protective effect of CH through MAPKs and NF-κB-signaling pathways in chondrocytes and the DMM OA model.

Currently, drugs for treating OA include NSAIDs (nonsteroidal anti-inflammatory drugs), corticosteroids, and DMOADs (disease-modifying osteoarthritis drugs), etc [15]. The most recently studied drugs are DMOADs, which have anti-inflammatory and chondroprotective effects as essential mechanisms by inhibiting factors involved in the OA developments [15]. However, MMP13 inhibitors (such as PG-116800) and ADAMTS inhibitors (such as GLPG1972/S201086), which showed very good efficacy in in vitro and in vivo studies, mostly failed to pass clinical trials due to lack of significant improvement efficacy or side effects [15,16,17]. Therefore, research on natural resources is being conducted to find materials that simultaneously exhibit anti-inflammatory and cartilage-protective effects. Several studies have been conducted to clarify the joint-protective and anti-arthritic efficacy of natural products. Olive and grape seed extract prevents post-traumatic osteoarthritis damage by inhibiting IL-1β-induced NO, PGE_2_, and MMP-13 expression [18], and wogonin (5,7-dihydroxy-8-methoxyflavone) protects cartilage by inhibiting the expression of inflammatory mediators (nitrite oxide, PGE_2_, COX-2, and iNOS) and ECM proteases (MMP-3, MMP-13, and ADAMTS-4) [19]. In addition, safflower seed extract protects cartilage by suppressing the expression of catabolic factors such as MMPs and ADAMTSs via blockade of the NF-κB-signaling pathway [20]. *Boswelia* extract protects joints by inhibiting the positive feedback that causes NF-κB and TNF-α to interact with each other [21].

The production of inflammatory factors is regulated by two different types of kinases; IκB kinases (IKKs) (Gamble et al., 2012) and mitogen-activated protein kinases (MAPKs). Upon binding of inflammatory cytokines to their receptors, IKKs induce phosphorylation of IκB, and the phosphorylated IκB is ubiquitinated and degraded via the proteasome [22]. The phosphorylation of IκB causes dissociation of the complex, translocating p65 into the nucleus, thereby promoting the transcription of inflammatory factors [19,22]. Also, in response to external stimuli such as inflammatory cytokines and oxidative stress, MAP3K and MAP2K are phosphorylated in sequence, and the final phosphorylation of MAPK ultimately induces various inflammatory and apoptotic responses [23,24,25]. Therefore, the inhibition of IKK- and MAPK-signaling pathways is a good suggestion to suppress inflammation and reduce the development of inflammatory diseases. Several studies have shown that activating the NF-κB- and MAPK-signaling pathways increases the expression of catabolic factors that disrupt joint cartilage, leading to OA progression [26]. Consistent with these findings, our study demonstrated that treatment with FeCH in IL-1β-stimulated chondrocytes resulted in the inhibition of JNK and p38 phosphorylation, alongside blocking the NF-κB-signaling pathway. Consequently, FeCH inhibits both MAPK and NF-κB activation in RC cells.

This study confirmed that IL-1β induced inflammation through increased expression of iNOS, PGE_2_, ADAMTS-4, -5, and MMP-1, -3, and -13 in RC. In addition, it was confirmed that pretreatment with FeCH inhibited IL-1β-induced phosphorylation of MAPKs (ERK1/2, JNK, and p38) and inhibition of nuclear translocation of NF-κB p65 subunit Based on these experimental results, it is suggested that FeCH has a cartilage protection effect by inhibiting the activity of pro-inflammatory cytokines and the activity of catabolic enzymes that degrade ECM through MAPKs and NF-κB-signaling pathways.

In an in vivo study, the DMM-induced OA model induces cartilage destabilization through medial meniscus damage, leading to loss of articular cartilage, and mimics human OA pathogenesis; therefore, the DMM model is widely used to investigate OA pathogenesis [27,28]. Orally administered CH extract protected cartilage in the DMM OA model through increased cartilage thickness and chondrocytes without any special adverse reactions. A notable result was that FeCH 50 mg/kg showed similar efficacy to celecoxib. Celecoxib is an NSAID drug used to reduce inflammation and pain in chronic diseases such as OA, and is also prescribed for patients with OA [15]. Therefore, if a future comparative analysis of the efficacy of the active ingredient of FeCH with celecoxib is conducted, the potential value of FeCH in improving joint efficacy is considered to be very high. In addition, *Ageratum conyzoides* L. extract inhibits the destruction and erosion of cartilage surfaces by inhibiting proteoglycan degradation in the DMM OA model [29]. When tissue sections of the DMM OA model used in this study were stained with fast green/safranin O, it was confirmed that the proteoglycans were distributed in the cartilage, and the degree of surface erosion increased with the concentration of FeCH. These results suggest that FeCH inhibits cartilage degradation and reduces articular damage in the knee joints of a DMM-induced OA model. These findings indicate that FeCH may be a useful material for developing osteoarthritis improvement agents.

Although the beneficial effects of FeCH on OA were demonstrated, this study had several limitations. The first is the monitoring period of in vivo experiments and the administered concentrations. Although this model induces OA through a similar surgical method as in humans, because OA is a chronic disease, the eight-week monitoring period may not be long enough to show long-term effects. Nevertheless, the 8-week period was also chosen in our previous study and was chosen based on protocols from other studies. However, further studies of 12 weeks or more are thought to be necessary to analyze long-term efficacy. Furthermore, there were only two concentrations used in the in vivo experiments, 25 and 50 mg/kg, which may be limited for expanding therapeutic potential. As can be seen from our study, 50 mg/kg showed similar efficacy to the normal group, but the therapeutic efficacy and toxicity at higher concentrations is not known. Therefore, studies of higher concentrations and longer duration suggest that further research into efficacy and safety is needed. Thirdly, only the DMM-induced OA model was used to evaluate the chondroprotective efficacy of FeCH. Because the causes of OA are diverse, including trauma, aging, genetics, and metabolic factors, the efficacy of FECH in chondroprotection cannot be generalized solely based on the effects of the post-traumatic OA development model used in this study. Therefore, studies in various models of OA are necessary to increase the value of FeCH for its chondroprotective effects. Finally, because this study did not use human-derived chondrocytes, there are limitations to fully understanding human cartilage physiology. If these limitations are overcome, the chondroprotective effect of FeCH is expected to become extremely robust.

## 4. Materials and Methods

### 4.1. Total Bioactive Compound Content Analysis

For total sugar content (TSC), CH was extracted at 85 °C for 25 min and filtered through a syringe filter (0.45 um, PTFE). Subsequently, 1 mL of 5% phenol solution and 5 mL of 95% sulfuric acid were added to the filtrate, stirred, and left in the room for 20 min. The absorbance was measured at 490 nm using an ultraviolet-visible spectrophotometer (UV-VIS Spectrophotometer). Sample measurements were performed in triplicates. A TSC calibration curve using a standard solution was prepared at 5–500 ug/mL with glucose as a standard material.

Total phenolic content (TPC) and 1 mL of CH extract solution were treated with 5 mL of Folin Ciocalteu reagent (10% *v*/*v*) and 2 mL for 5 min. Next, Na_2_CO_3_ (7% *w*/*v*) was added. The mixture was allowed to stand at room temperature for 1 h, and the absorbance was measured at 765 nm using a UV-VIS Spectrophotometer at 765 nm. The TPC was determined using a calibration curve with a gallic acid (GA) standard solution.

Total flavonoid contents (TFC): 0.5 mL of CH extract solution was added sequentially to 1.5 mL of MeOH, 10% aluminum nitrate (Al(NO_3_)_3_·9H_2_O) solution, and 0.1 mL each of 1M potassium acetate solution (CH_3_COOK). Then, 2.8 mL was added, stirred sufficiently, and incubated for 40 min, and the absorbance was measured at 415 nm using a UV-VIS spectrophotometer. TFC was determined using a calibration curve using a quercetin (QUE) standard solution.

Total carotenoid content (TCC): The CH extract was added to a solvent mixture of hexane, EtOH, acetone, and toluene in a ratio of 10:6:7:7. This was followed by ultrasonic extraction for 1 h at 60 °C, and then filtering through a membrane filter (0.45 um, PTFE). The absorbance was measured at 446 nm using a UV-VIS Spectrophotometer, and the total carotenoid content of each sample was analyzed by applying the lutein extinction coefficient.

### 4.2. FeCH

*C. hypnaeoides* was collected from Jeollanam-do Wando, washed twice, and dried. The extraction solvent used was edible alcohol (named “fermented alcohol”). Thus, 100% fermented alcohol was supplied from mainstream supplier and mixed with sterilized water to make 30% before use. *C. hypnaeoides* was extracted using 30% fermented ethanol and concentrated, freeze-dried, and stored at 4 °C until used in the experiment. Fermented ethanol extracts of *C. hypnaeoides* (FeCH) were dissolved in a complete growth medium (DMEM F12 containing 10% FBS and 1% penicillin/sterptomycin,c/dmem/f12 media) at 2 mg/mL concentration.

### 4.3. Preparation of Reagents

The Live/Dead™ Viability/Cytotoxicity kit was purchased from Thermo Scientific (Waltham, MA, USA), and 3-(4,5-dimethylthiazol-2-yl)-2,5-diphenyltetrazolium bromide (MTT) and 4′,6-diamidino-2-phenylindole (DAPI) were purchased from Sigma-Aldrich (St. Louis, MO, USA). The following antibodies were purchased from various suppliers: iNOS (#15323), COX-2 (#179800), TNF-α (#66579), IL-6 (#229381), ADAMTS-5 (#182795), ADAMTS-4 (#185722), and MMP13 (#39012) from Abcam (Cambridge, UK); COX-1 (#4841S), MMP-3 (#143515), p-ERK (#9101S), ERK (#9102S), p-JNK (#4668S), JNK(#9252S), p-p38 (#4631S), and p38 (#9212S) from Cell Signaling Technology (Danvers, MA, USA); MMP-1 (#352518) from LSBio (Seattle, WA, USA); α-tubulin (#32-2500), collagen type II (#12789), and p65 (#16545) from Invitrogen (Waltham, MA, USA); Lamin (#56144) from Santa Cruz Biotechnology (Dallas, TX, USA).

### 4.4. Rat Primary Chondrocyte Isolation and Culturing

To isolate chondrocytes, the method of Jang et al. (2022) was used with slight modifications [30]. Five-day-old Sprague-Dawley rats were purchased from Damool Science (Daejeon, Republic of Korea), and after sacrificing the experimental animals, only two cartilages were extracted. Extracted cartilage was digested with 0.3% (*w*/*v*) collagenase type II dissolved in DMEM/F12 at 37 °C for 16 h. The enzyme solution was filtered through a cell strainer (0.45 μm). Chondrocytes were seeded at 2 × 10^6^ cells/mL into 6- or 12-well cell culture plates with DMEM/F12 containing 10% FBS and 1% penicillin/streptomycin in a humidified incubator with 5% CO_2_ at 37 °C. The chondrocytes were cultured up to 90% confluency and were not passaged during the experiment.

### 4.5. Cell Viability Assay

The survival rate of primary rat chondrocytes (RC) in response to FeCH was analyzed using MTT assay. Cells were seeded at 1.5 × 10^6^ RC per well in a 12-well cell culture dish and cultured overnight. Then, 0.25, 0.5, 1, and 2 mg/mL FeCH were allowed to react for 24 h. After the reaction, 100 µL of MTT (5 mg/mL) solution was added, and the mixture was allowed to react at 37 °C, 5% CO_2_, and incubated for 2 h. After the reaction, the culture medium was removed, formazan salt was dissolved in DMSO, and cell viability was measured at 590 nm using an ELISA reader (Epoch; BioTek Instruments, Winooski, VT, USA). The absorbance of the control group was set at 100%, and the absorbance of the experimental group treated with the sample was expressed as a percentage (%) compared with that of the control group.

### 4.6. Measurement of Nitric Oxide

RC was pretreated with FeCH for 1 h prior to treatment with IL-1β for 24 h. The supernatants were collected. The accumulation of NO in the culture medium was determined from the quantity of released nitrite (NO_2_) and the stable oxidation product of NO, using Griess reagent (1% [*w*/*v*] sulfanilamidein, 0.1% [*w*/*v*] naphthyl ethylenediamine in 5% [*v*/*v*] phosphoric acid, and DW).

The culture medium (100 mL) was mixed with an equal volume of Griess reagent, and the absorbance of the mixture was measured at 540 nm using a microplate reader (Epoch Bioteck, Bio-Tek Instruments Inc. Winooski, VT, USA). The quantity of nitrite was determined by comparison with a sodium nitrite standard curve.

### 4.7. Westernblot Analysis

RC were pretreated with FeCH for 1 h before being treated with IL-1β for 24 h. Total protein was extracted using PRO-PREP (iNtRON Biotechnology, Seongnam-si, Republic of Korea) and quantified using a bovine serum albumin (BSA) standard (Thermo Scientific, Rockford, IL, USA). Proteins (16 μg) were separated by 8% or 10% SDS-PAGE and transferred onto a polyvinylidene difluoride membrane (Bio-Rad Laboratories, Hercules, CA, USA). The membranes were blocked with 5% BSA in Tris-buffered saline containing 0.1% Tween 20 (TBST) for 50 min.

Subsequently, the membranes were incubated overnight with a specific primary antibody at 4 °C, followed by three washings with TBST. After washing, the membranes were incubated with horseradish peroxidase-conjugated secondary antibodies for 50 min. The bands were detected using an enhanced chemiluminescence (ECL) kit (Millipore, Bedford, MA, USA) and visualized using an image reader (Ras-4000, Fujifilm, Tokyo, Japan).

### 4.8. ELISA

RC were pretreated with FeCH for 1 h prior to treatment with Il-1β for 24 h. The production of TNF-α and PGE_2_ was analyzed using the culture medium according to the ELISA assay protocol.

### 4.9. Alcian Blue Stain

RC were seeded at a density of 2 × 10^5^ cells/mL in a 6-well culture plate. Cells were pretreated with FeCH and treated with IL-1β for 24 h. The cells were fixed with 70% ethanol at room temperature for 1 h and rinsed thoroughly with PBS. After that, the cells were dyed in 1% alcian blue solution for 24 h, dissolved in 6M guanidine-HCL, and the absorbance was measured at 620 nm using an ELISA reader (Epoch; BioTek instruments, Winooski, VT, USA).

### 4.10. Immunocytochemistry (ICC)

RC (3 × 10^5^ cells/well) were cultured in 4-well slides and allowed to attach to the bottom of the chamber for 3 days. After fixing with 4% paraformaldehyde for 10 min, the cells were washed three times with 1X PBS. Next, 0.1% Triton X-100 was added to permeabilize the cells for 5 min. The cells were washed three times with 1X PBS and blocked with 1% BSA (ABC kit, horse) for 30 min. Anti-collagen type II was added and the mixture was incubated at 4 °C for 24 h. After washing three times with PBS, the secondary antibody was incubated at room temperature for 1 h in the dark. The cells were then washed three times with PBS in a light-shielded space. Five minutes after adding the DAPI solution, images were captured using a fluorescence microscope (Eclipse TE2000; Nikon Instruments, Tokyo, Japan).

### 4.11. DMM-Induced OA Models in Rats

Damool Science (Daejeon, Republic of Korea) provided male 6-week-old SD rats subjected to a one-week stabilization period. OA rats were generated by surgical destabilization of the knee joint’s medial meniscus (DMM).

The rats were randomly divided into five groups (*n* = 4) and orally administered FeCH or celecoxib, which are anti-arthritis drugs, daily for 8 weeks from the day after surgery: group 1 (control), group 2 (DMM), group 3–5 (DMM, 25 and 50 mg/kg, FeCH). All rats were sacrificed on the day after the final administration.

### 4.12. Histology Evaluation and Score

Safranin O and fast green staining were performed to evaluate proteoglycan loss in articular cartilage tissues. Knee joint samples were fixed in 10% NBF, decalcified with 0.5M EDTA (PH7.4), dehydrated through a series of ethanol solutions, and embedded in paraffin blocks. After that, lateral serial sections were cut into 8 μm sections and stained with safranin O and fast green. OA severity was assessed using the Osteoarthritis Research Society International (OARSI) score.

### 4.13. Statistical Analyses

All experiments were independently repeated three times and the results were expressed as standard deviation values. The significance between each experimental group was tested using Dunnett’s test and analysis of variance (ANOVA) in GraphPad Prism software (version 5.0) and the results were considered satistically significant when the *p*-value was less than 0.05.

## Figures and Tables

**Table 1:** TSC, TPC, TFC, and TCC of CH extracts.

TSC	TPC	TFC	TCC
166.77 ± 1.3	197.2 ± 1.5	59.5 ± 0.8	1.16 ± 0.06

## Data Availability

All data generated or analyzed during this study are included in this manuscript and its information files.
